# Comparative transcriptome analysis reveals the phosphate starvation alleviation mechanism of phosphate accumulating *Pseudomonas putida* in *Arabidopsis thaliana*

**DOI:** 10.1038/s41598-023-31154-1

**Published:** 2023-03-25

**Authors:** Sonal Srivastava, Manish Ranjan, Nasreen Bano, Mehar Hasan Asif, Suchi Srivastava

**Affiliations:** 1 Division of Microbial Technology, CSIR-National Botanical Research Institute, Rana Pratap Marg, Lucknow, 226 001 India; 2 Academy of Scientific and Innovative Research, AcSIR, Ghaziabad, 201002 India; 3 Computational Biology Laboratory, Genetics and Biotechnology Division, CSIR-National Botanical Research Institute, Rana Pratap Marg, Lucknow, 226 001 India

**Keywords:** Microbiology, Molecular biology, Plant sciences

## Abstract

Phosphate starvation is one of the major factors limiting plant productivity globally. Soil microflora with an inherent trait of phosphate accumulation directly influences soil phosphorus level by regulating its labile form in soil solution. However, the detailed mechanism involved during their interaction with plants under phosphate deficient conditions is still unexplored. Hence, to dissect these complex gene regulatory networks, transcriptome analysis of *A. thaliana* roots grown under phosphate starved conditions in presence of phosphate accumulating bacteria (*Pseudomonas putida;* RAR) was performed. Plants grown under phosphate starved conditions showed upregulation of phosphate starvation responsive genes associated with cell biogenesis, stress, photosynthesis, senescence, and cellular transport. Inoculation of RAR upregulated genes linked to defense, cell wall remodeling, and hormone metabolism in stressed plants. Gene ontology analysis indicated the induction of S-glycoside, glucosinolate, and glycosinolate metabolic processes in RAR inoculated plants under phosphate stressed conditions. Further, protein–protein interaction analysis revealed upregulation of root development, cation transport, anion transport, sulfur compound metabolic process, secondary metabolic process, cellular amino metabolic process, and response to salicylic acid in RAR inoculated plants under phosphate starved conditions. These results indicate the potential role of phosphate accumulating bacteria in alleviating phosphate starvation in plants by involving multiple pathways.

## Introduction

Phosphorus (P) is the second most important plant nutrient and its limitation severely affects the plant’s performance and productivity. Precipitation of P fertilizers with calcium, iron, and aluminium in soil restricts their availability to plants^1^**.** Phosphate availability immensely affects plants’ physiology, metabolism, and crop performance. The fully oxidized form of inorganic phosphate (Pi) is vital for the photosynthesis process, thereby, known to play a key role in regulating energy conservation and assimilation^2^. It is involved in an array of metabolic processes including energy metabolism, macro-molecular biosynthesis, photosynthesis, glycolysis, enzyme activation/inactivation, redox reactions, signaling, and carbohydrate metabolism^3–5^. Additionally, it also affects the chemical stability and cellular retention of biological macromolecules and metabolites.

Plants grown under limited phosphate conditions evolve multiple adaptive responses, together termed as phosphate stress responses (PSRs). It involves coordination and integration of local and long distance signaling which promotes enhanced acquisition of Pi along with remobilization within plants^6^. Multiple signaling molecules mediate the establishment of PSRs, among which Pi itself is a primary signal which further evokes other molecules viz. sugar, hormones, metabolites, reactive oxygen species, and peptides^7,8^. Plants adapt to Pi deficient conditions by modulating different morphological and metabolic adaptations such as changes in root system architecture, anthocyanin accumulation, galactolipid synthesis, the release of organic acids, phosphatases, and nucleases mediated through differential gene expression^9–11^. In *A. thaliana*, PHOSPHATE STARVATION RESPONSE 1 (*PHR1*) and its closely related transcription factors cumulatively referred to as *PHR* transcription factors are the master regulator of Pi sensing and signaling^12^. In addition, *PHR1* also promotes the expression of NITRATE-INDUCIBLE GARP-TYPE TRANSCRIPTIONAL REPRESSOR1 (*NIGT1*) family genes resulting in reduced nitrate uptake. Therefore, *PHR1* involve in two different transcriptional cascades forming a link between the regulation of both nitrogen and phosphorus^13^. In *A. thaliana*, Pi starvation and oxygen deficiency contain a set of overlapping genes and these responses are also under the control of *PHR1*^14^. Besides *PHR1*, another transcription factor viz. *WRKY*, *zinc finger, R2R3 MYB, MYB* like*, ERF* and *G2* like families also play a salient role in the regulation of PSRs^15–20^.

Despite being abundantly present, the unavailability of Pi in the soil is a major concern. Plant growth promoting rhizobacteria (PGPR) has the potential of mobilizing soil bound Pi through solubilization and mineralization. Soil microbes also accumulate P within their biomass which approximately accounts for 2 to 10% of total soil and can exceed up to 50%^21,22^. These phosphate accumulating microbes efficiently compete with plants for available orthophosphate from soil solution which represents the temporarily unavailable immobilized pool of P for plants. This form of P becomes available to plants over time, therefore, P immobilization is an important mechanism for maintaining P supply in soil solution^23,24^. Stress tolerant microbes along with their P solubilization abilities are known to play a pivotal role in stress mitigation in plants^25^. In a previous study, we demonstrated the efficacy of polyphosphate accumulating bacteria (PAB) in plant growth promotion and salinity stress alleviation in *A. thaliana*^26^.

The interaction between plant and PGPR is not an outcome of gene for gene interaction, indeed it is a multigene response^27^. Numerous gene based studies have been performed using phosphate solubilizing bacteria in plants under stress conditions. However, interaction mechanism of PAB with plants under phosphate starved condition is yet to be explored. Therefore, elucidation of complex gene regulatory networks regulating the interaction between plant and PAB will unravel many aspects for alleviating phosphate deficiency, a common but precarious constraint to the agricultural system. Hence, to get into the molecular insight underlying the interaction between PAB and plants, the transcriptome analysis of *A. thaliana* roots grown under phosphate starved conditions was performed. Furthermore, the study was validated through qRT-PCR using selected candidate genes belonging to different metabolic activities.

## Results

### Polyphosphate accumulation and stress tolerance potential of RAR

**Figure 1:**
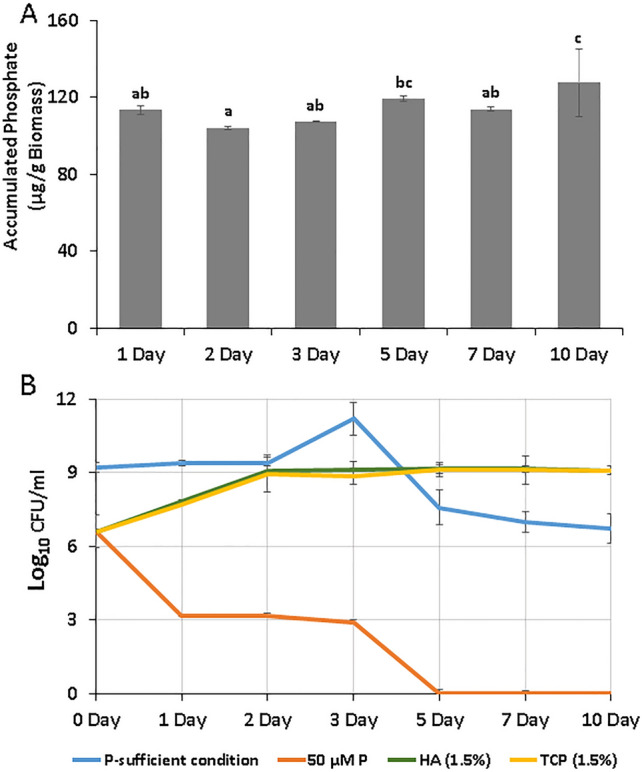
Accumulated phosphate within *Pseudomonas putida* biomass (**A**); Effect of different phosphate sources [(Normal P (0.3% KH_2_PO_4_ + 1.2%Na_2_HPO_4_), limited P (50 µM KH_2_PO_4_) and unavailable P (1.5% hydroxyapatite and tricalcium phosphate] on growth of *Pseudomonas putida* (**B**).

Phosphate accumulating potential and stress tolerance (different P sources) of *Pseudomonas putida* (RAR) was evaluated for 10 consecutive days. Results showed approximately similar accumulated phosphate content in RAR till the 7^th^ day (Fig. 1A). Significantly higher phosphate accumulation was observed on the 10^th^ day in RAR. In presence of unavailable sources i.e. HA and TCP, RAR extended its growth phase and survived up to the 10^th^ day of incubation. However, RAR doesn’t survive under 50 µM P (KH_2_PO_4_) condition and attained the death phase on the 5^th^ day of incubation (Fig. 1B).

### RAR inoculation modulates physicochemical parameters in *A. thaliana* under phosphate starved condition

The efficacy of P accumulating RAR on the growth of *A. thaliana* plants subjected to phosphate stress was evaluated (Supplementary Fig. 1). Compared to uninoculated plants, significantly higher root length, number of rosette leaves, siliques, and dry weight was observed in RAR inoculated plants (Supplementary Table 2). Phosphate stress (HA; unavailable P) resulted in declined shoot length, dry weight, and siliques of *A. thaliana* plants which were enhanced in RAR  treated plants by 30.34%, 78.57%, and 48.55%, respectively. Additionally, lowered P content in phosphate starved plants was enhanced in presence of RAR under stress conditions. The dry weight of the plants grown under limited P (available 50 µM P) was higher compared to control conditions. Additionally, a higher reduction in P content was evident in plants grown under HA conditions as compared to 50 µM available P conditions. Therefore, based on the results HA source of P was selected for further study.

### RAR modifies root system architecture of *A. thaliana* under phosphate starved condition

Effect of RAR on root system architecture was studied through in vitro interaction of *A. thaliana* with RAR under normal and P starved conditions. Results showed improved growth of the plant treated with RAR as compared to the control (Supplementary Fig. 2A). Treatment of RAR improved the shoot growth and root branching (Supplementary Fig. 2A,B). Furthermore, microscopy of roots also revealed root hair formation under phosphate starved conditions in both RAR inoculated and uninoculated plants as compared to the control (Supplementary Fig. 2A,B). Since the effect of phosphate starvation was more prominent on roots, therefore, in the present study root tissue was selected for transcriptome analysis.

### Summary of transcriptome sequencing and mapping onto *A. thaliana* reference genome

**Figure 2:**
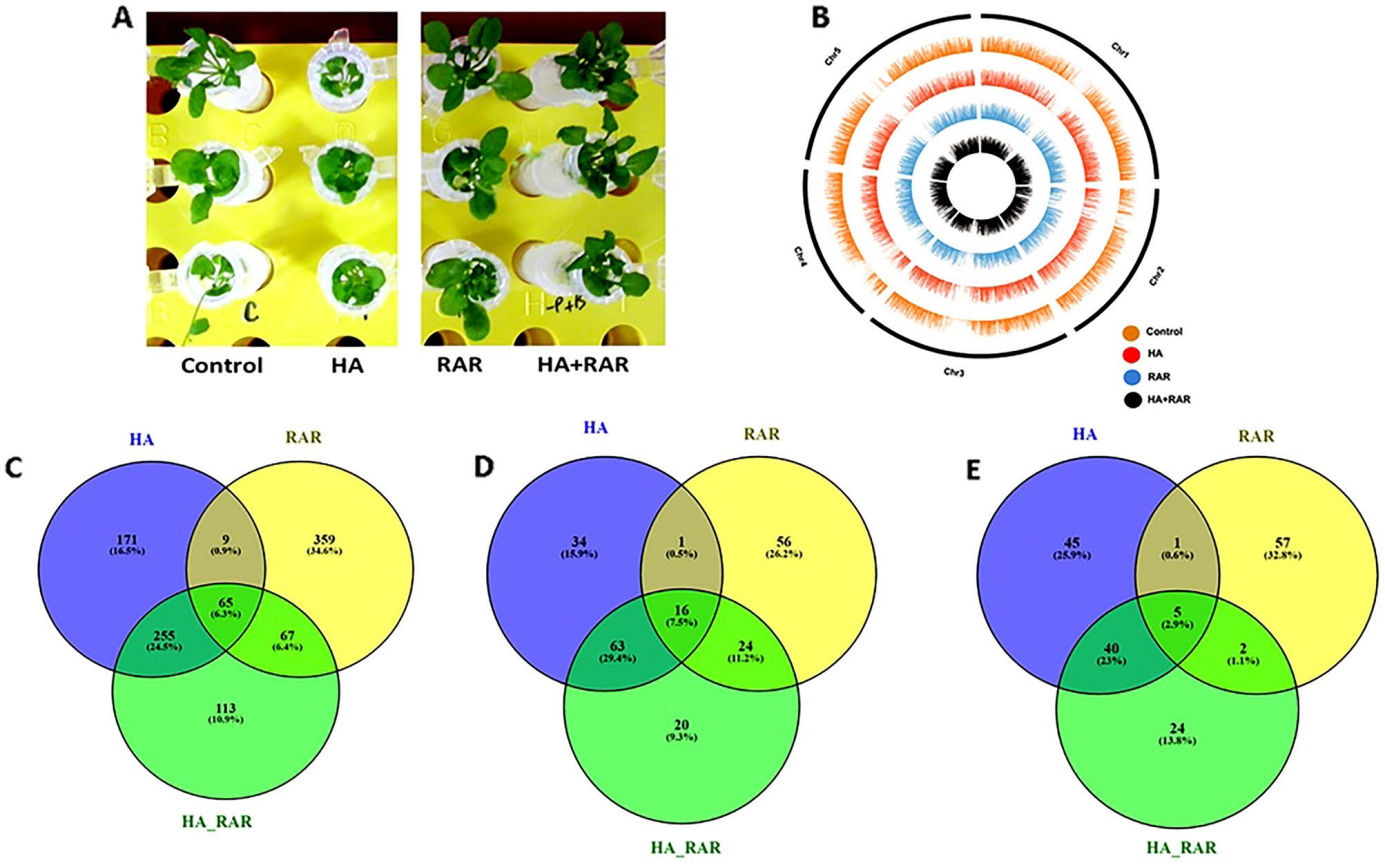
Growth of *Arabidopsis thaliana* in presence of RAR under phosphate starved condition in the hydroponic system (**A**); Circos showing the histogram of genes mapped onto 5 chromosomes of *Arabidopsis thaliana* (**B**). Venn diagram showing total number of expressed transcripts (**C**); Venn diagram of the differentially expressed upregulated (**D**) and downregulated genes (**E**) in RAR, HA and HA + RAR treatment as compared to control.

For transcriptome analysis plants were grown under *in-vitro* condition in the hydroponic system (Fig. 2A). RNA extracted from *A. thaliana* roots was used for Illumina sequencing and data was processed for the generation of high quality reads and bases (Table 1). All 8 libraries of control (1,2), RAR (1,2), HA (1,2), and HA + RAR (1,2) produced an average of 15.88 million reads in the range of 11.64 to 18.36 million for each sample. The total number of reads, total bases, and data generated through transcriptome sequencing are given in Table 1.

**Table 1:** Read statistics of transcriptome of different samples.

S. no.	Sample	No. of reads	Total bases	Data obtained (Gb)
1	Control 1	11,644,447	1,753,837,222	~ 1.75
2	Control 2	17,513,170	2,637,161,861	~ 2.64
3	RAR 1	17,459,025	2,628,351,214	~ 2.63
4	RAR 2	18,365,698	2,764,142,720	~ 2.76
5	HA 1	15,577,803	2,345,735,641	~ 2.35
6	HA 2	16,568,894	2,494,141,219	~ 2.49
7	HARAR 1	14,864,128	2,237,235,998	~ 2.24
8	HARAR 2	15,110,846	2,274,381,789	~ 2.27

Differentially expressed genes plotted on circos plot showed gene alteration in all 5 chromosomes of *A. thaliana* (Fig. 2B)*.* Venn diagram was plotted and analyzed to identify the differentially expressed genes (DEGs) between different treatments of HA, RAR, and HA + RAR (Fig. 2C–E). Overall analysis revealed that 65 DEGs were found to be common between all three treatments (Fig. 2C). Furthermore, the Venn diagram showed 9 common DEGs between HA and RAR. Results also showed overlapping of 255 and 67 DEGs between HA-HA + RAR and RAR-HA + RAR treatments, respectively. Whereas, a total of 171, 359, and 113 unique DEGs were preferentially expressed in HA, RAR, and HA + RAR treatments, respectively. Out of 1039 DEGs obtained, 114 genes were upregulated in HA, 97 in RAR, and 123 in HA + RAR treatments as compared to control (Fig. 2D). On the contrary, a lesser number of genes were found to be downregulated in HA (91), RAR (65) and HA + RAR (71) treatments (Fig. 2E).

### Differential expression of genes under all growth conditions

Differential expression of genes based on *P* value < 0.05 was compared in plants grown under HA, RAR, and HA + RAR treatments as compared to the control (Supplementary appendix 1,2,3). Plants inoculated with RAR showed upregulation of genes involved in different metabolic processes such as hormonal metabolism, cell wall modification, defense activities, and transportation. Expression of gibberellic acid-stimulated *Arabidopsis* (*GASA*) gene i.e. *GASA3* (*At4g09600*) and *GASA5* (*At3g02885*) was highly induced in *A. thaliana* roots inoculated with RAR. The study also showed upregulation of genes associated with abscisic acid signaling viz. ABA-induced transcription repressor 5 (*AITR5*; At5g50360), highly ABA-induced *PP2C* gene 1 (*HAI1*; *At5g59220*), highly ABA-induced *PP2C* gene 2 (*HAI2*; *At1g07430*), HOMEOBOX 12 (*HB*-*12*; *At3g61890*) and ethylene signalling i.e. ethylene responsive factor54 (*ERF54*; *At4g28140*) specifically in the RAR inoculated plants. Role of RAR in cell wall remodeling through overexpression of lipid transfer protein 3 (*LTP3*), delta 9 desaturase 1 (*ADS1*; *At1G06080*), polygalacturonase abscission zone *A. thaliana* (*PGAZAT*; *At2G41850*), 3-ketoacyl-coa synthase 3 (*KCS3*; *At1G07720*), expansin A10 (*EXPA10*; *At1g26770*), xylogen protein 1 (*XYP1*; *At5g64080*) and osbp (oxysterol binding protein)-related protein 4B (*ORP4B*; *At4g25850*) was also evident in the study. Interestingly some transporters such as high affinity nitrate transporter 2.6 (*NRT2.6*; *At3g45060*), nramp metal ion transporter 6 (*NRAMP6*; *At1g15960*) were found to be upregulated in RAR inoculated plants. Expression of defense associated genes viz. myo-inositol oxygenase 4 (*MIOX4*; *At4G26260*), galactinol synthase 2 (*GolS2*; *At1G56600*), ascorbate peroxidase 5 (*APX5*; *At4g35970*) and beta glucosidase 24 (*BGLU24*; *At5g28510*) was also noted in plants treated with RAR. Results showed prominent expression of putative cytochrome P450 (*At3g26200*) and embryonic cell protein 63 (*ECP63*; *At2g36640*) in presence of RAR.

Plants subjected to phosphate deprived conditions showed higher induction of genes such as senescence-associated gene 12 (*SAG12*; *At5g45890*), usually multiple acids move in and out transporters 19 (*UMAMIT19*; *At1g21890*), *UMAMIT17* (*At4g08300*), 2-oxoglutarate (*2OG*) and Fe(II)-dependent oxygenase superfamily protein (*At2g44800*), oxidoreductase 2OG-Fe(II) oxygenase family protein (*At2g48080*), cysteine/histidine-rich C1 domain protein (*At2g37805*), gibberellic acid methyltransferase 2 (*GAMT2*; *At5g56300*), fantastic four 2 (*FAF2*; *At1g03170*), dehydration response element B1A (*DREB1A*; *At4g25480*), small auxin upregulated 72 (*SAUR72*; *At3g12830*), phosphoenolpyruvate carboxylase kinase 2 (*PPCK2*; *At3g04530*) and glutathione s-transferase 14 (*GSTF14*; *At1g49860*).

Overexpression of genes associated with nitrate metabolism and transportation viz. nitrate transporter 2.2 (*NRT2.2*; *At1g08100*), *NRT2.5* (*At1g12940*), *NRT1* (*At3g21670*), glutamate dehydrogenase 3 (*GDH3*; *At3g03910*) and glutamine synthetase 1;4 (*GLN1*;4; *At5g16570*) was upregulated in HA and HA + RAR treatment. Phosphate stress responsive genes such as *IPS1* (*At3g09922*) and phosphate transporter 2 (*PHT1*;2; *At5g43370*) were overexpressed in HA and HA + RAR treatment, however, the expression was more prominent in phosphate starved plants inoculated with RAR (HA + RAR). Phosphate starvation-induced gene 2 (*PS2*; *At1g73010*) was expressed in all three conditions, while, repressed expression was observed in RAR inoculated plants. Besides, sulfate [*Sultr1*;3 (*At1g22150*), *Sultr2;1* (*At5g10180*)] and nitrate [*NRT2:1* (*At1g08090*), *NPF3.1* (*At1g68570*), transporters also showed overexpression in all the three treatments.

### Differential expression analysis of *A. thaliana* genes under phosphate starved condition

**Figure 3:**
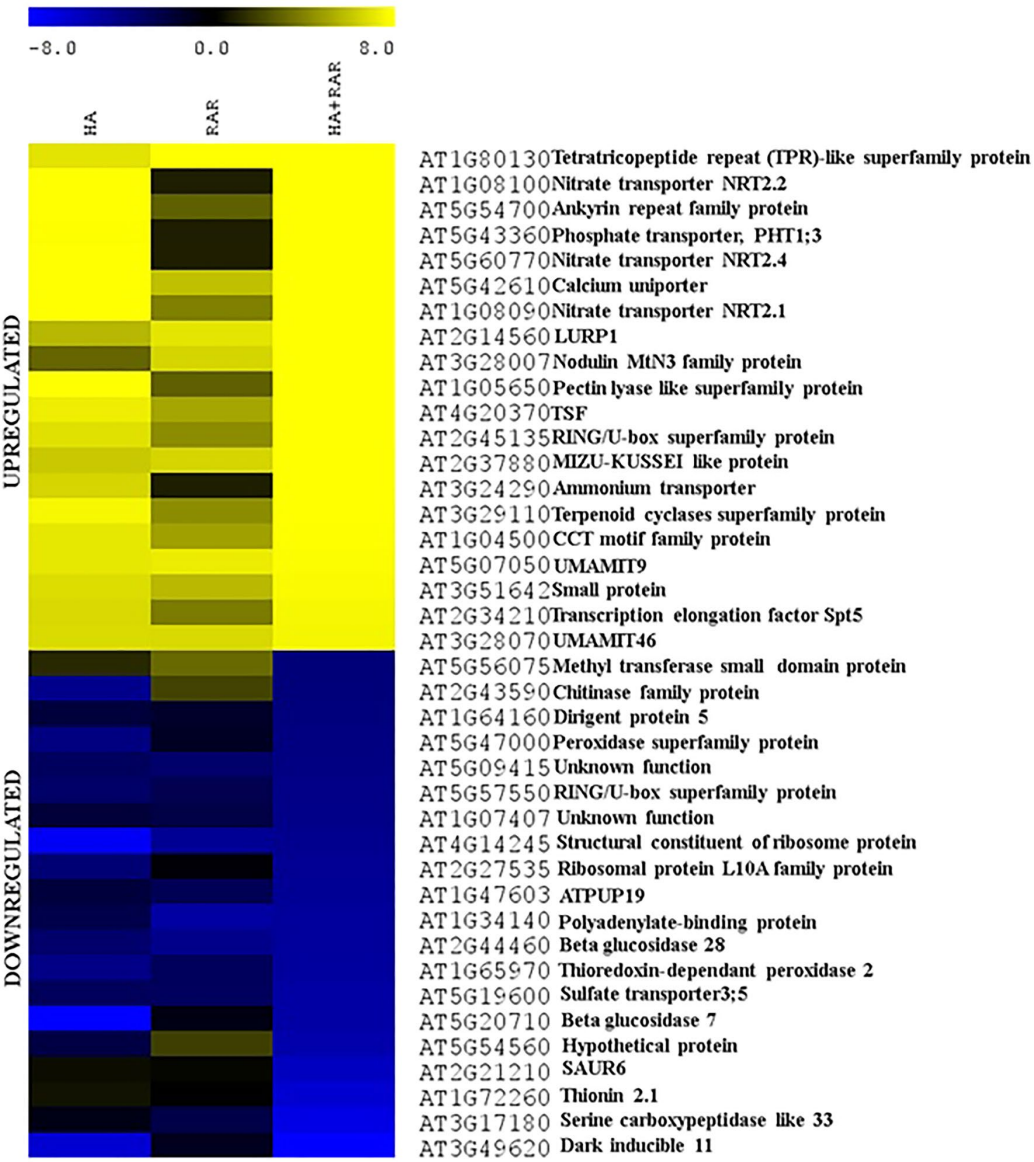
Differential expression of top 20 upregulated and downregulated genes in *Arabidopsis thaliana* roots grown under phosphate deprived condition in presence of *Pseudomonas putida*.

A heat map of the top 20 upregulated and downregulated genes in HA, RAR, and HA + RAR treatments as compared to non-inoculated control was generated (Fig. 3). Inorganic phosphate transporter *PHT1;3* involved in transmembrane transport of phosphate was found to be upregulated in HA and HA + RAR treatment among which the expression was higher in RAR treated plants under Pi starved condition. A study showed the upregulation of nitrate transporters (*NRT2.2, NRT2.4,* and *NRT2.1*) in HA and HA + RAR treatment, however, its expression was not evident in RAR inoculated plants. In addition, results showed upregulation of ammonium and amino acid transporter (*UMAMIT9* and *UMAMIT46*). Sulfate transporter was downregulated in all three growth conditions as compared to the control. Nodulin *MtN3* family protein involved in sugar transmembrane transportation was upregulated in the RAR inoculated plants under both P-sufficient and starved conditions. However, the gene was not expressed in phosphate starved plants. Tetratricopeptide repeat (*TPR*)-like superfamily protein involved in oxidative stress management was highly upregulated in RAR and HA + RAR treatment, however, its repressed expression was noted in plants grown under P stress condition. *SAUR6* and *Thionin* *21* was expressed only under phosphate starved condition, whereas, it was downregulated in RAR inoculated treatments. Results showed the downregulation of peroxidase family protein, *ATPUP19*, thioredoxin peroxidase 2, dark inducible 11,beta glucosidase 28 and polyadenylate binding protein genes in all three treatments.

### Comparative expression analysis of genes associated with plant growth promotion, root system architecture, defense metabolism, phytohormones, and phosphate transport

Expression analysis of genes belonging to different processes including root system architecture, defense metabolism, phytohormones, phosphate transport and plant growth promotion was studied. Most of the genes associated with plant growth promotion were found to be upregulated in presence of RAR (Table 2). Genes specifically upregulated in RAR inoculated plants included anthranilate synthase (*At5g05730*), *IAA1* (*At4g14560*), *WRKY60* (*At2g25000*), *WRKY70* (*At3g56400*), amino acid biosynthetic pathway and nutrient uptake (*At5g63890*) and IAA production during plant–microbe interaction (*At4g36110*). In addition, genes *WAK1* (*At1g21250*), hormone synthesis (*At3g23890*), and mitotic and meiotic division (*At5g20850*) were upregulated in both RAR and HA + RAR treatments. Genes associated with carbohydrate metabolism (*At1g05030*), jasmonic acid *LOX2* (*At3g45140*), glutathione-s-transferase (*At5g17220*), and ethylene biosynthesis (*At4g26200*) showed upregulation in HA and HA + RAR treatment. However, these genes were either downregulated or not expressed in RAR alone treatment.

**Table 2:** Log fold change value of genes associated with plant growth promotion, root system architecture, defense metabolism, phytohormones, and phosphate transport in HA, RAR, and HA + RAR treatments.

	Locus identifier	Gene	HA	RAR	HA + RAR
			Log fold change		
Plant growth promotion	AT5G05730	ASA1, anthranilate synthase 1	− 0.60502	0.233222	− 1.19295
	AT4G14560	IAA1	− 0.45933	1.245183	− 0.31336
	AT2G25000	WRYK60	− 0.11835	0.67778	0.178139
	AT3G56400	WRKY70	− 1.00746	0.907445	− 1.36474
	AT1G21250	WAK1	0.320022	3.180495	1.194565
	AT3G45140	jasmonic acid (JA) LOX2	1.612366	− 1.81294	1.884971
	AT1G05030	Carbohydrate metabolism	0.174888	− 0.33848	0.290582
	AT1G06730	Plastid nucleoside kinase	0.123424	− 0.08761	0.056039
	AT1G53730	STRUBBELIG-receptor family 6	0.025972	0.15064	0.160056
	AT1G12890	Transcription factor	− 0.27459	1.217637	0.556539
	AT1G01260	Transcription factor	0.140424	− 0.24861	− 0.11045
	AT5G63890	Histidinol dehydrogenase	0.100673	0.302311	0.037689
	AT3G23890	Topoisomerase II	0.511329	1.067717	1.130213
	AT2G46370	Jasmonate-amido synthetase	0.022017	− 0.02026	0.01576
	AT5G20850	RAD51	0.563679	1.213952	1.086052
	AT4G36110	SAUR9	− 1.23748	0.222742	− 2.10603
	AT5G17220	Glutathione S-transferase	2.265442	2.553179	2.069202
	AT1G74930	ERF/AP2 transcription factor family	1.081301	0.158679	1.217591
	AT4G26200	Ethylene biosynthesis genes	0.36849	− 0.70514	0.486748
Root system architecture	AT1G79700	AP2/ERF-type transcriptional activator	− 1.33638	0.487252	− 0.13305
	AT1G23010	Low phosphate root 1	0.132059	0.587806	0.432539
	AT4G28610	PHR1	0.161888	0.028235	− 0.06149
	AT3G03710	Chloroplast polynucleotide phosphorylase	0.03284	− 0.19015	0.399371
	AT3G25710	Helix-loop-helix transcription factor	0.074142	− 0.61421	− 0.39981
	AT5G21040	F box protein	0.091412	− 0.09987	− 0.04077
	AT5G42810	Inositol-pentakisphosphate 2-kinase	0.335513	0.328126	0.4272
	AT3G20630	Ubiquitin-specific protease	− 0.02404	− 0.11438	0.093818
	AT1G27740	Root hair defective like	− 0.16931	0.158572	− 0.16314
	AT1G13620	Root meristem growth factor− 2	0.383748	1.090409	0.669725
	AT2G04025	Root meristem growth factor− 3	0.561811	0.888145	1.084354
	AT5G60810	Root meristem growth factor− 1	1.036245	1.525388	1.825072
	AT3G07360	E3 ligase 9	0.190537	0.042808	0.027918
	AT1G65800	Receptor Kinase 2	− 0.24397	0.69488	0.542802
	AT5G21040	F box motifs	0.091412	− 0.09987	− 0.04077
	AT1G66470	Root hair defective 6	0.372088	0.977157	1.129053
	AT1G01380	ETC1	1.357817	− 0.46166	0.557949
Defense	AT3G12500	Basic chitinase	− 0.79084	0.580785	− 0.61569
	AT2G43590	Putative endochitinase	− 4.26441	2.12599	− 3.67553
	AT3G49120	Putative peroxidase	− 1.41458	0.499832	− 0.9197
	AT5G64100	Putative peroxidase	− 1.15536	0.835064	− 0.52223
	AT4G19810	Chitinase	− 0.05348	0.337477	0.176733
	AT1G72260	Thionin	0.627182	− 0.00836	− 6.34483
	AT3G50970	Dehydrin Xero2	− 0.08879	0.755893	− 0.48026
	AT4G25780	Putative pathogenesis-related protein	1.644367	0.672576	1.285413
	AT5G47910	Respiratory oxidase oxidase protein	0.468684	− 1.20605	0.162101
	AT5G39580	Peroxidase	− 0.77574	1.265149	− 0.70767
	AT5G39720	Avirulence responsive protein	1.358477	1.700944	2.177848
	AT5G17220	Glutathione S-transferase, putative	2.265442	2.553179	2.069202
	AT3G47540	Chitinase	− 0.29663	0.75535	− 0.44857
	AT1G19200	Senescence-associated protein	1.467621	1.319232	1.296234
	AT3G09940	Monodehydroascorbate reductase	0.813197	− 0.67246	0.325767
	AT1G69080	Universal stress protein	0.879286	− 0.41984	0.797882
	AT1G08830	superoxide dismutase	0.26508	− 0.26706	0.026401
Phytohormones	AT1G68320	MYB62	− 0.91856	0.254571	0.016459
	AT5G13080	WRKY75	− 1.41862	0.111279	− 1.72453
	AT1G68320	MYB62	− 0.91856	0.254571	0.016459
	AT2G47190	MYB2	0.430055	− 1.44589	0.386696
	AT3G62980	AtTIR1, Transport inhibitor response	0.111033	0.27616	0.469989
	AT1G19220	Auxin response factor 19	0.731804	− 0.84521	0.568221
	AT2G27050	Ethylene-insensitive3-like1	0.087121	0.276828	0.233629
	AT2G01830	Cytokinin response 1	− 0.0748	0.270271	0.243102
	AT5G67030	Zeaxanthin epoxidase	0.1306	− 0.02059	0.188658
	AT4G26080	ABA insensitive 1	− 0.34884	0.677509	− 0.07961
	AT5G57050	ABA signal transduction	− 0.10187	1.476148	0.468082
Phosphate transporters	AT2G38940	PHT1;4; Inorganic phosphate transporter	1.574022	0.534836	1.95555
	AT5G20150	SPX domaincontaining proteins	3.249601	1.643516	3.192507
	AT2G26660	SPX domaincontaining proteins	0.907458	0.416665	0.911567
	AT1G68740	PHO1	1.962392	0.154712	1.761031
	AT5G43360	PHT1;3	7.743493	0	9.22805
	AT3G47420	Glycerol-3-P transporter	2.725013	0.968898	2.280317
	AT5G43370	PHT1;2	4.763233	0.20171	5.615784
	AT3G52190	PHF1	1.362951	0.529231	1.240638
	AT2G32830	PHT1;5	1.930059	1.704908	1.366535
	AT5G54800	Glucose-6-phosphate/phosphate translocator	0.135257	0.188966	− 0.0262
	AT5G43370	PHT2	4.763233	0.20171	5.615784
	AT5G43350	PHT1;1	1.397895	− 0.60161	1.491866
	AT1G76430	PHT1;9	0.196505	− 0.78779	0.289093
	AT1G20860	PHT1;8	2.288292	1.475084	2.466618
	AT1G61800	PT2	0.768295	5.129416	4.814035
	AT2G32830	PHT1;5	1.930059	1.704908	1.366535

Plants grown under phosphate starved conditions showed upregulation of disease resistance response protein-related (*At1g22900*), thionin (*At1g72260*) and respiratory burst oxidase protein (*At5g47910*) linked to defense activities in plants (Table 2). However, inoculation of RAR resulted in downregulation of these genes in plants under phosphate starved condition (HA + RAR). RAR inoculation upregulated the expression of defense associated genes including [basic chitinase (*At3g12500*), putative endochitinase (*At2g43590*), putative peroxidase (*At3g49120*, *At5g64100*), dehydrin xero2 (*At3g50970*), peroxidase *ATP24a* (*At5g39580*) and chitinase (*At3g47540*)], while, these genes were either downregulated or not expressed in other two treatments.

Genes associated with modification in root system architecture were differentially expressed in HA, RAR, and HA + RAR treatments (Table 2). Plants grown under phosphate starved conditions showed upregulation of *PHR1* (*At4g28610*) and U box/armadillo repeat-containing E3 ligase9 (*AtPUB9*; *At3g07360*). Root meristem growth factor (*RGF2; At1g13620*), *RGF3* (*At2g04025*), *RGF1* (*At5g60810*), root hair defective 6 (*At1g66470*) and, *ipk* (*At5g42810*) were upregulated under all growth conditions. Among all, the gene involved in root hair patterning (*ETC1*; *At1g01380*) was upregulated in HA and HA + RAR treatment. RAR induced the expression of ethylene insensitive-3 like 1 (*At2g27050*), auxin receptor (*At3g62980*) and ABA signal transduction (*At5g57050*) encoding genes directly involved in induction and development of root hair under P starved condition.

High affinity phosphate transporters belonging to *PHT1* family viz. *PHT1;1* (*At5g43350*), *PHT1*;*2* (*At5g43370*), *PHT1;3* (*At5g43360*), *PHT1;4* (*At2g38940*), *PHT1*;5 (*At2g32830*), and *PHT1*;*8* (*At1g20860*) were upregulated in HA and HA + RAR treatments (Table 2). These transporters are responsible for Pi acquisition and mobilization in plants under starved conditions. Among these, *PHT1*;*5* (Pi homeostasis) and *PHT1;8* (root to shoot translocation of orthophosphate) were also expressed in RAR inoculated plants. Phosphate transporter (*PT2*; *At1g61800*) identical to *AtPT2* was specifically upregulated in RAR and HA + RAR treatment depicting role of inoculum in mediating expression of particular transporter for P uptake.

### Gene ontology and Kyoto encyclopedia of genes and genomes pathways analysis of differentially expressed genes

**Figure 4:**
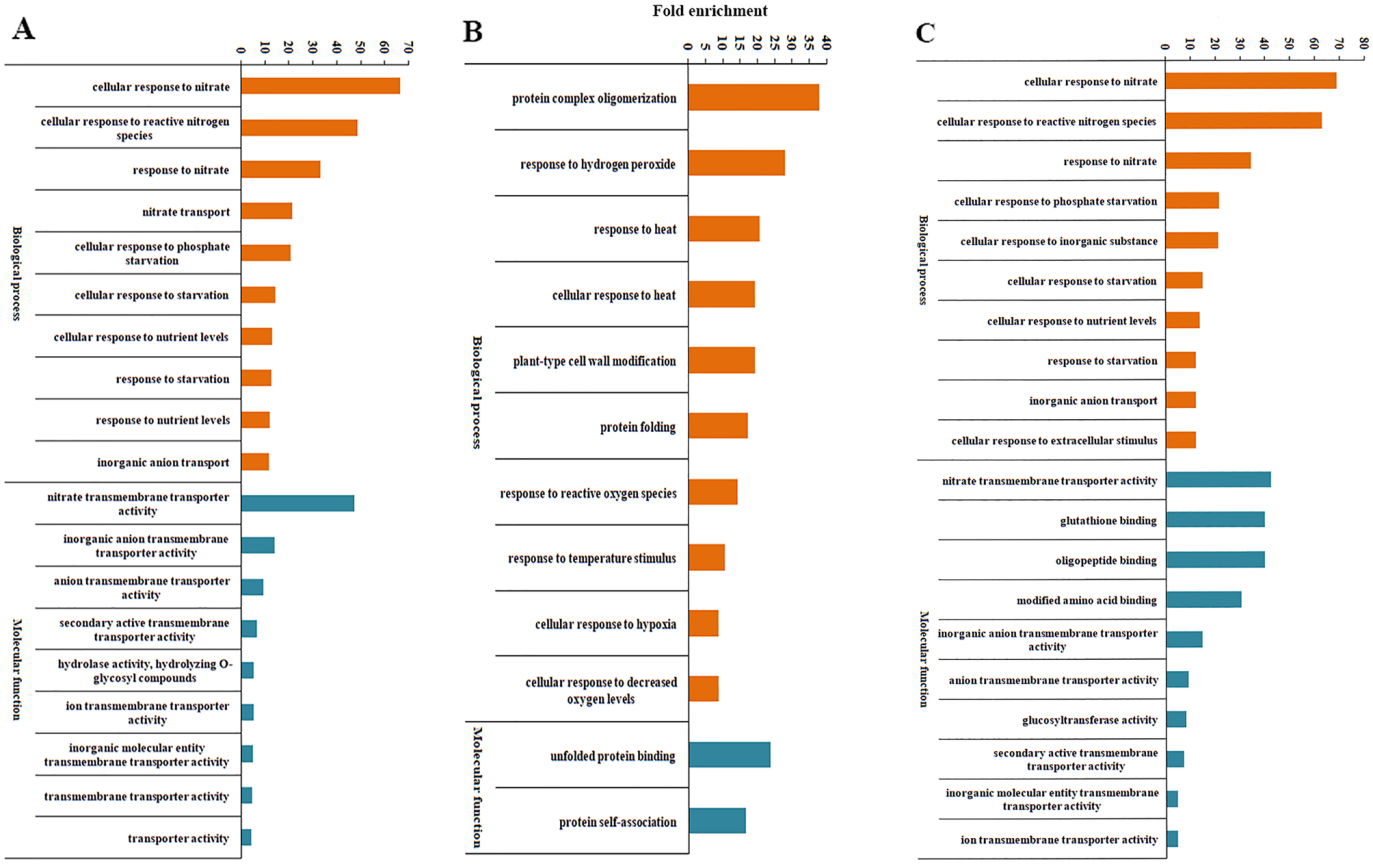
Gene ontology analysis of genes differentially expressed in HA (**A**) RAR (**B**) and HA + RAR (**C**) treatment. Gene ontologies were categorized by their significance.

We identified different biological processes (BP) categories enriched in treatments HA, RAR and HA + RAR (Fig. 4A–C; Supplementary Table 3). Top overrepresented categories were most common in HA and HA + RAR treatments. These processes were associated with response to nitrate stress, reactive nitrogen species, phosphate starvation, nutrient levels, detoxification, toxic substance and secondary metabolic processes. BP categories uniquely enriched in HA treatment included nitrate transport, aging and ion transport. RAR inoculated plants exhibited BP associated with protein complex oligomerization, hydrogen peroxide, heat, plant cell wall modification, reactive oxygen species, temperature, oxidative stress, salt stress, osmotic stress, water deprivation, abscisic acid and abiotic stimulus. BP categories such as S-glycoside, glucosinolate, glycosinolate metabolic process were specifically enriched in phosphate starved plants inoculated with RAR (HA + RAR).

GO molecular function (MF) categories enriched in HA, RAR and HA + RAR treatments is represented in Fig. 4A–C and Supplementary Table 3. Common MF categories in HA and HA + RAR treatment included nitrate, inorganic anion, anion, ion and inorganic molecular transmembrane transporter activity. MF specifically enriched in HA + RAR treatments constituted glutathione binding, oligopeptide binding, oligopeptide binding and glucosyltransferase activity. However, RAR treated plants showed enrichment of MF associated with unfolded protein binding and protein self-association.

### Functional enrichment analysis of clusters

**Figure 5:**
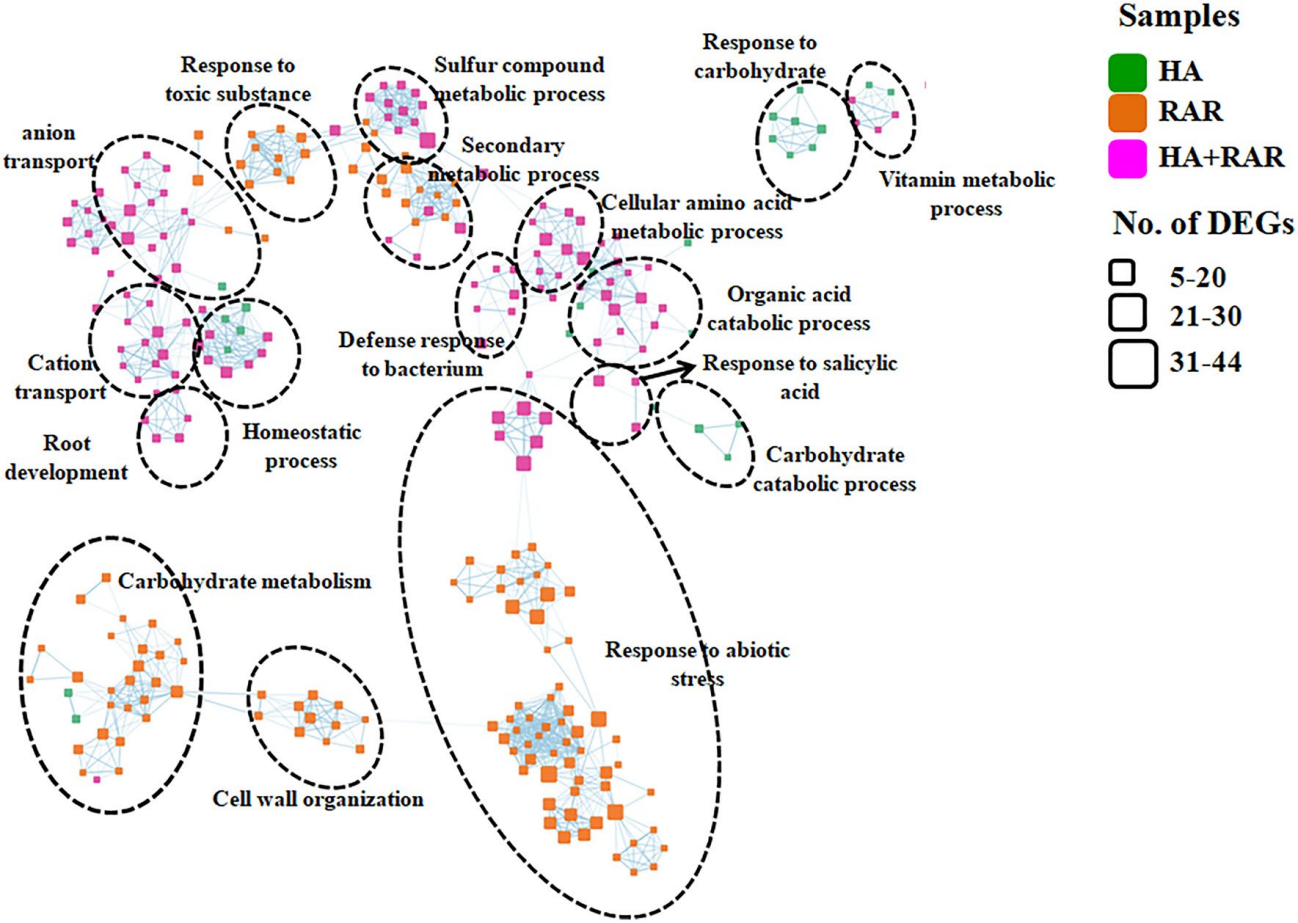
Functional enrichment analysis of the HA, RAR and HA + RAR treatments and its interactome analyses uncovers functional significance in these three samples. Node colour represent the different sample such as green, orange and pink colour showing the HA, RAR and HA + RAR samples. The size of the nodes shows the number (setSize) of differentially expressed genes identified in enrichment.

Protein–protein interaction (PPI) analysis showed presence of eleven significant clusters in the plants grown under different growth conditions viz. RAR, HA and HA + RAR (Fig. 5). Among all three treatments, the majority of DEGs were evident in HA + RAR treatments. These DEGs were associated with various processes such as root development, cation and anion transport, sulfur compound metabolic process, secondary metabolic process, cellular amino metabolic process and response to salicylic acid. RAR inoculated plants showed involvement of DEGs belonging to carbohydrate metabolism, cell wall organization, response to toxic substance and abiotic stress. Secondary metabolic process and response to abiotic stress was common in both RAR and HA + RAR treatments. Plants grown under phosphate starved condition showed enrichment of DEGs associated with response to carbohydrate.

### Validation of differentially expressed genes through real time qPCR

**Figure 6:**
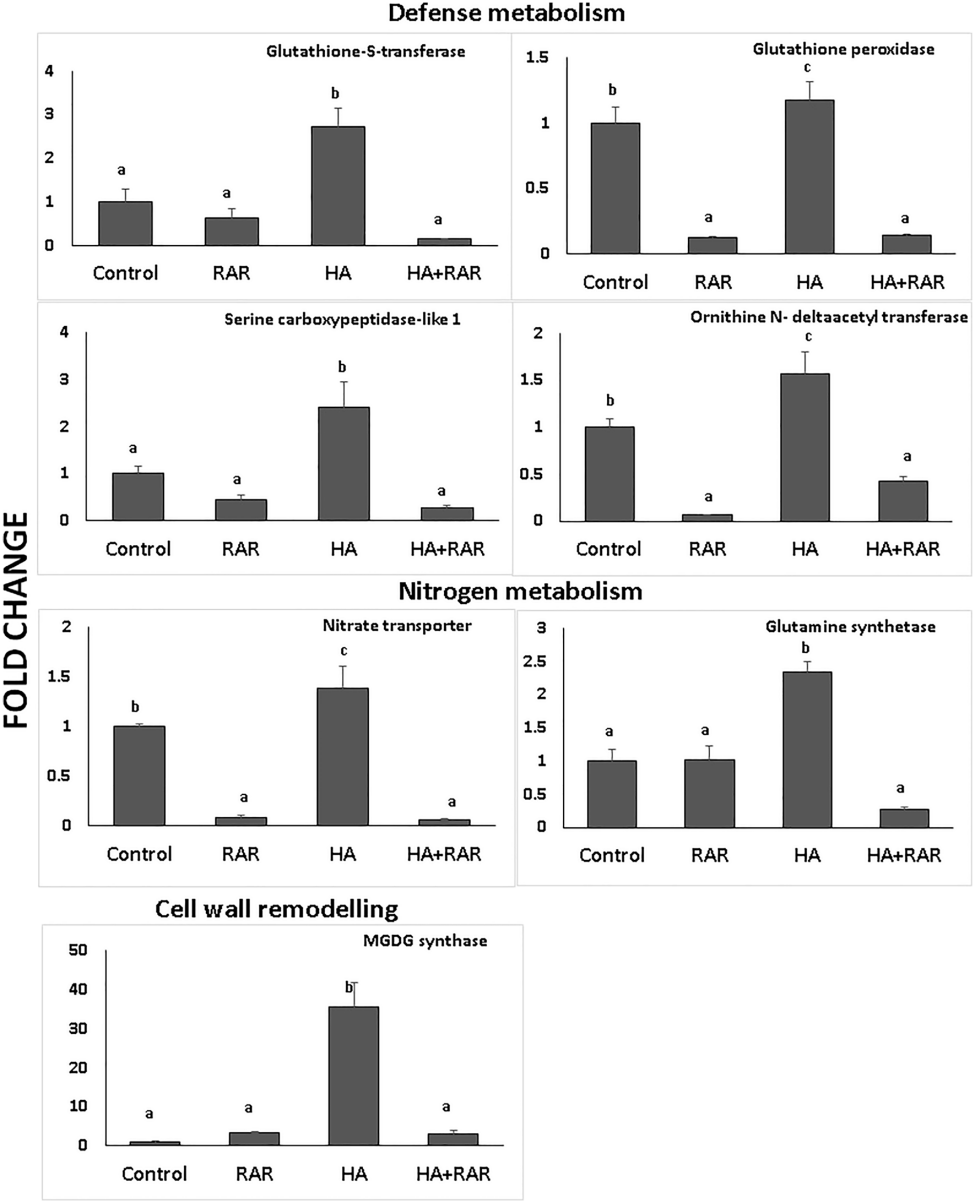
Effect of phosphate accumulating *Pseudomonas putida* inoculation on expression of genes associated with defense metabolism, nitrogen metabolism and cell wall remodelling in *Arabidopsis thaliana* roots grown under phosphate starved condition.

Overall transcriptome analysis revealed that phosphate starvation affected the defense metabolism, nitrogen metabolism, and cell wall remodelling in plants. Henceforth, differential expression of genes associated with these metabolic processes was assessed. Expression of genes associated with defense metabolism viz. glutathione S-transferase [*GST*; *At1g78340*.*1*], glutathione peroxidase [*GPX*; *At4g31870*.*1*], serine carboxypeptidase-like 1 [*At5g36180*.*1*] and ornithine N-delta-acetyltransferase [*At2g39030*.*1*] were significantly upregulated in *A. thaliana* under P starved condition as compared to control. On the contrary, their reduced expression was evident in the presence of RAR (Fig. 6). Elevated expression of genes associated with nitrogen metabolism i.e. nitrate transporter (*At1g08090.1*) glutamine synthetase gene (*At5g16570.2*) was found in the *A. thaliana* grown under P starved condition, however, inoculation of RAR significantly reduced the expression of both genes under stress condition. Similarly, genes involved in glycolipid biosynthesis [monogalactosyldiacylglycerol synthase (*At5g20410.1*)] was upregulated by ~ 35 under stress condition, while, significantly reduced expression was evident in RAR treated plants (RAR and HA+RAR). The results obtained were consistent with the transcriptome data indicating the reliability of high throughput NGS technologies for gene expression studies (Supplementary Fig. 3).

## Discussion

**Figure 7:**
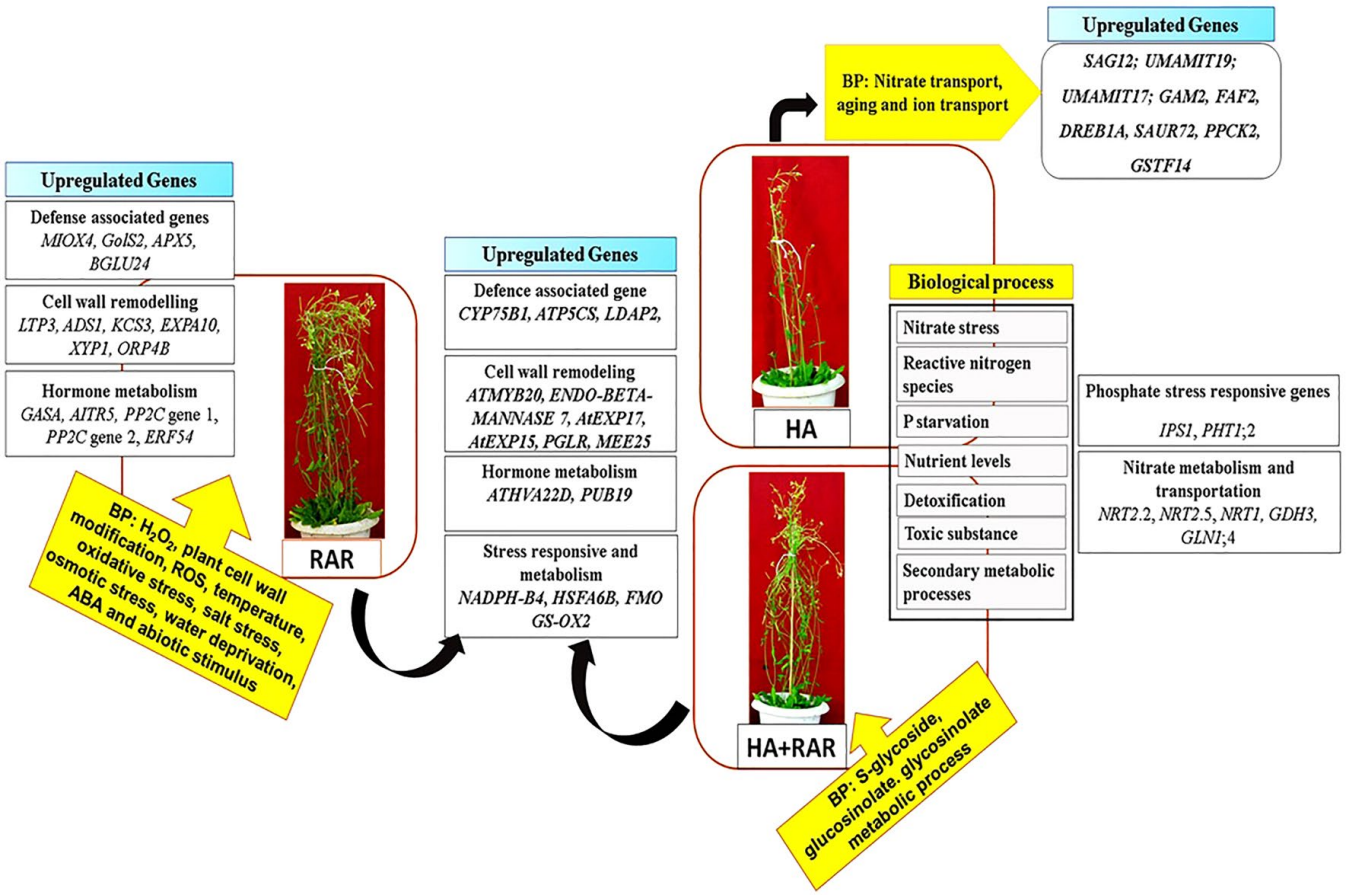
A figure demonstrating the summary of overall modulations in different processes in *Arabidopsis thaliana* grown under phosphate starved condition in presence of phosphate accumulating *Pseudomonas putida.*

The study involves the elucidation of detailed molecular mechanisms occurring during the interaction of polyphosphate accumulating bacteria *P. putida* (RAR) with *A. thaliana* ecotype *Col-0* grown under phosphate deprived conditions. Transcriptome analysis using RNA-Seq has been used to analyze genes that are differentially regulated by Pi deprivation and inoculation of RAR. Environmental factors substantially regulate the developmental program of root. Phosphate deprivation resulted in induced root hair formation under in vitro conditions in the present study. Increased density of root hairs enable the plants to meet the nutrient demand of the plant under inadequate supply^28,29^. RAR mediated enhanced root hair formation under both normal and stressed conditions is as per the earlier report^30,31^. Phosphorus acquisition by plants depends on a concerted action of a range of physiological and morphological adaptations which involve numerous signaling pathways^32^. Transcriptome analysis revealed differential expression of genes associated with the different processes in *A. thaliana* grown under different growth conditions viz. HA, RAR and HA + RAR. Modulations in different processes in plants grown in presence of RAR under phosphate starved condition is summarized in Fig. 7.

### *P. putida* mediated altered metabolism, growth, and development of *A. thaliana* under phosphate starved condition

Phosphate starvation responsive genes are linked with multiple processes such as cell biogenesis, cellular transport mechanism, amino acid metabolism, response to stresses, photosynthesis, senescence, and others^33^. Present study also showed upregulation of different genes associated with these functions in plants. Upregulation of senescence associated gene (*SAG12*) under phosphate starved condition is in accordance to earlier report^34^. Pi starved condition in plants represses the photosynthesis resulting in carbon starvation which activate proteins and amino acid catabolism for release of free ammonium^33^. Present study showed the upregulation of usually multiple acids, in and out transporter genes (*UMAMIT17* and *UMAMIT19*) responsible for amino acid transportation in plants.

Transcriptome analysis revealed enrichment of GO functions associated with transportation viz. phloem transport, nucleocytoplasmic transport, nuclear transport and vascular transport in RAR inoculated plants. These functions are involved in transportation of water, metabolites, sugar, constitutive nuclear proteins, import/export of key signalling molecules which is essential for growth, development, hormone signalling and responses to environmental stimuli^35,36^. In addition, RAR inoculated plants showed enrichment of indole glucosinolate biosynthetic process. As per earlier reports, indole glucosinolates are metabolically associated with auxin homeostasis in plants and its disruption negatively affect the plant growth and development^6,37^. Enrichment of pathways associated with stress regulation in the plants grown under P deprived condition in presence of RAR shows its involvement in stress management in plants under P deficient condition. Gene ontology study showed abundance of biological functions associated with the hypoxic conditions in the plants grown under phosphate deprived condition. The overlapping of transcriptional response to phosphate and oxygen deficiency leads to the induction of set of commonly induced genes which is under control of transcription factor phosphate starvation response1 (*PHR1*)^14^. Involvement of C_2_H_2_-type zinc finger like protein (*At2g28710*) in present study demonstrates its role in regulation of Pi starvation as reported earlier^17^.

### *P. putida* mediated modulation in phytohormones in *A. thaliana* under phosphate starved condition

Phytohormones are known to regulate various biological processes linked to plant growth and stress response cascades^38^. Plants inoculated with RAR showed upregulation of numerous genes associated with hormone regulation such as gibberellic acid (*GASA3* and *GASA5*), abscisic acid (*AITR5*, *HAI1* and *HAI2*) and ethylene signalling (*ERF54*). Abscisic acid and gibberellins are well associated with plants developmental processes involving seed dormancy, root growth, flowering time and seed maturation^38^. Additionally, interaction of ethylene with other hormones regulates both growth and senescence in plants^39^. Auxin responsible for altering root system architecture under phosphate starvation condition^40^ was majorly upregulated in the plants treated with HA. *SMALL AUXIN UP RNAs* (*SAURs*) are early auxin response genes which regulate the growth and development of plants^41^. Present study showed upregulation of SAUR genes viz. *SAUR6*, *SAUR41* and *SAUR72* in plants subjected to phosphate starvation condition indicating the involvement of these genes under nutrient deprived condition.

### *P. putida* mediated modulation in transporters under phosphate starved condition

Uptake of nutrient from soil is pivotal for plants’ growth and development which is achieved by a set of specialized transporters. These transporters are involved in sensing and radial transportation of water and nutrients to vascular tissues^42^. Present study showed modulations in transporters linked with different nutrient uptake in plants. Upregulation of nitrate transporters in HA and HA + RAR treatment observed in present study has also been demonstrated earlier that the role of nitrate transporters in regulating phosphate starvation response in plants^43^. High-affinity phosphate transporter 2 (*PHT1;2*; *At5g43370*) responsible for external inorganic phosphate uptake was overexpressed under both HA and HA + RAR conditions. Higher expression in HA + RAR as compared to HA treatment suggest the involvement of RAR in enhanced uptake of Pi under starved condition. CC-type glutaredoxins are expressed in plants to mediate signalling under nitrate deprived condition^44^. However, present study reports the involvement of CC-type glutaredoxin (*ROXY*) family (*At5g11930*) in plants grown under phosphate starved condition.

### *P. putida* mediated management of reactive oxygen species in *A. thaliana* under phosphate starved condition

Significant accumulation of reactive oxygen species is being reported under phosphate deprived condition^30^. Genes associated with quenching of reactive oxygen species i.e*.* glutathione S-transferase was upregulated in phosphate starved plants as per earlier reports^45^. *DREB1* gene activates the *AtTPPF* transcription which regulates the level of reactive oxygen species, trehalose and sucrose in plants during drought stress^46^. Present study also showed the upregulation of *DREB1* gene under phosphate starved condition.

Gene expression study through qPCR revealed the activation of defense metabolism in *A. thaliana* under phosphate starved condition evident through higher expression of glutathione peroxidase, glutathione S-transferase, serine carboxypeptidase-like 1 and ornithine N-delta acetyl transferase. Lower expression of these genes in the presence of RAR reveals the availability of P to the plants under starved condition along with important role of PAB in regulating P homeostasis. Henceforth, the present study provides a detailed molecular mechanism of phosphate accumulating RAR mediated phosphate stress alleviation in *A. thaliana*. Numerous modification in metabolic pathways in RAR inoculated plants under phosphate starved condition indicates the substantial role of PAB in regulating P homeostasis in plants. Present study is the first report encompassing a regulatory network involve during the interaction of phosphate accumulating bacteria with *A. thaliana* under phosphate starved conditions. This progress will be beneficial in consideration of phosphate accumulation as an important trait of microorganisms for mediating P availability and regulating stress response in plants under P deficient conditions.

## Material and methods

### Characterization of phosphate accumulating *P. putida*

*P. putida MTCC 5279,* (RAR) already characterized as abiotic stress tolerant plant growth promoting bacterial strain^47^, with an ability to promote the growth of model plant *A. thaliana* under P starved salinity stressed conditions^30^. In present study, RAR was further characterized for phosphate accumulating potential and its growth in presence of different P sources. For phosphate accumulation, RAR was inoculated in NBRI-PA medium comprised of available form of P source i.e. disodium hydrogen orthophosphate (Na_2_HPO_4_) and potassium dihydrogen phosphate (KH_2_PO_4_) and accumulated Pi was determined upto 10 days. Inoculated NBRI-PA media was incubated at 28 °C for 48 h and bacterial cells were harvested by centrifugation at 10,000 rpm for 10 min at 4 °C. Extraction of Pi from bacterial cells was performed as described earlier by^48^. Extracted Pi was estimated by molybdenum blue method as described earlier^49^. Further, growth of RAR under varied P conditions viz. sufficient and deficient (50 µM KH_2_PO_4_, 1.5% HA and TCP) was evaluated by monitoring their growth followed by their viable cell count determination (CFU/ml) at different time intervals upto 10 days.

### *In-vitro* interaction of *P. putida* with *A. thaliana* under phosphate starved condition

To evaluate the plant growth promoting potential of *P. putida*, its interaction with *A. thaliana* was performed on solid MS medium (Murashige and Skoog medium; 0.5x) and pot (soil rite) condition. Further, to elucidate the mechanism of interaction, a plant growth experiment was set up in a closed hydroponic system.

*A. thaliana* plants were grown on solid MS media plate supplemented with 1% sucrose (pH adjusted to 5.6) and 0.8% agar (solidifying agent). Surface sterilized pre-germinated one week old seedlings of *A. thaliana* was transferred in MS media plates in a single row containing available and unavailable source of P. P-starved condition was created by adding 500 µM of hydroxyapatite (HA) as unavailable phosphate source in MS media^50^. RAR was streaked at the bottom of the plates.

For pot experiments, surface sterilized seeds of *A. thaliana* was sown in soil rite as described earlier^30^. Log phase grown culture of RAR was inoculated around the roots and after 20 days phosphate starvation condition was created by supplying unavailable P (HA; 500 µM) and available limited P (50 µM P) to the plants. Treatments included control, RAR, HA, HA + RAR, 50 µM P and 50 µM P + RAR. Plants were harvested after 2 weeks of stress and root length, shoot length and dry weight of plants were recorded. Phosphate content was estimated in *A. thaliana* plants grown under pot conditions according to Tsvetkova and Georgiev (2003).

Further, for hydroponic experiment, surface sterilized pre-germinated seedlings of *A. thaliana* (*Col-0*) were transferred in liquid ½ MS media supplemented with 1% sucrose (pH adjusted to 5.6) in micro-centrifuge tubes. After 4 days, plants were inoculated with log phase grown culture of *P. putida* RAR (@1%, spun and resuspended in 20 mM magnesium sulphate, O.D. ~ 0.6). After 5 days, plants were transferred to the new micro-centrifuge tubes containing MS media supplemented with either available P source (KH_2_PO_4_) or unavailable source (500 µM of HA) and again RAR was inoculated in the plants. Treatments in the experiment were control (CONT), PAB (RAR), 500 µM of HA and HA + RAR. After 8 days of phosphate stress, root tissue of the plant was excised, washed and stored at − 80 °C for transcriptome study. This study involve the experiment based on *Arabidopsis thaliana* which comply with institutional, national, and international guidelines and legislation.

### Illumina sequencing and mapping

Total RNA was extracted from the roots of hydroponically grown *A. thaliana* under different growth conditions viz. CONT., HA, RAR and HA + RAR. RNA was extracted using the ZR plant RNA miniprep (ZYMO Research) kit using the manufacturer’s protocol. The RNA sequencing library was prepared using TruSeq stranded mRNA library prep kit as per the manufacturer's protocol. The sequencing was done on the Illumina NextSeq500 platform to generate 2 × 75 bp reads. The sequencing data was generated using the fastaq format. The quality of the raw data was checked using FastQC^52^. Further, to eliminate the low quality reads Trimmomatic 0.36 tool was used^53^. These filtered and high quality reads were used for further analysis. The high quality reads were mapped onto the *A. thaliana* reference genome using the HISAT2 software^54^ using default parameters. After mapping, the resulting files were sorted and converted to BAM files using standard SAMtools^55,56^ followed by annotation and abundance estimation using the Stringtie program^57^.

### Differential gene expression and GO analysis

The differential gene expression analysis was carried out using the edgeR package of the R studio^58^. The differential expression of the treated samples were carried out in comparison to the untreated control (RAR *vs* Cont, HA *vs* Cont, HA + RAR *vs* Cont). The differential genes were filtered based on P-value < 0.05 and a log_2_ fold change ≤  − 1 or ≥ 1. The differentially expressed genes in all three treatments were used for the plotting of heat maps using the MeV tool^59^. Based on the annotation from the TAIR database the differentially expressed genes were functionally categorized. Further, the differentially expressed genes selected from previous reports belonging to different processes viz. plant growth promotion, root system architecture, phytohormones, defense, and phosphate transportation in plants were visualized using the MapMan 3.5.1 software^60^. To know the function of genes, the GO annotations of the differentially expressed genes were fetched from the *Arabidopsis* information resource (TAIR) database using the bulk retrieval tool. The GO annotations were used for plotting the functional categories viz. biological processes, molecular functions, and cellular components. The GO enrichment analysis was done using the String database^61^. Further, the WebGestalt toolkit^62^ was used to identify the top ranking genes in each of the treatments.

### Protein–protein interaction (PPI) network analysis

The sets of genes with log2FC ≥ 2 for up-regulated and ≤ -2 for down-regulated genes with Pvalue ≤ 0.05 were selected for functional enrichment study (FES). These putative enriched functions were analyzed and visualized in Cytoscape version 3.9.0^63^ using the enrichment map module.

### Availability of supporting data and accession number

The raw reads data is available at the NCBI Sequencing Read Archive (SRA; SUB11641967) database (Bioproject: PRJNA851914; https://www.ncbi.nlm.nih.gov/bioproject/?term=PRJNA851914).

### Quantitative real time PCR analysis

Real time qPCR analysis of randomly selected genes from the study was performed for validation of results. Total RNA was extracted from hydroponically grown *A. thaliana* root tissue using RNA easy mini kit (Qiagen) according to the manufacturer’s instructions. DNase enzyme treated RNA was used for cDNA preparation through revertaid H minus cDNA synthesis kit (Thermo). Real time PCR was carried out with Quanti-Tect TM SYBR® Green PCR kit (Qiagen) on Stratagene Mx3000P systems with a 10 μl reaction system. The reaction mixture comprised of forward and reverse primer (0.5 μl each of 10 μM concentration), 5 μl SYBR green master, and cDNA. Cycle conditions included a preliminary step at 95 °C for 10 min, 40 cycles of denaturation and amplification at 94 °C for 30 s, 55 °C for the 30 s, and 72 °C for 30 s. Fold change was calculated from ct value by the delta-delta ct method. Each sample was analyzed in triplicate. The primer pairs used in the study are provided in Supplementary Table 1.

### Statistical analysis

One-way analysis of variance (ANOVA) was performed to identify the significantly different treatments using SPSS 16.0.
